# Trends in the Dispensing of Oral Anti‐Cancer Medications Across Australia Over 10 Years

**DOI:** 10.1002/pds.70126

**Published:** 2025-03-25

**Authors:** Michael James Leach, Emily Griffin, Sinead Hickmott, Holly Atkinson, Louise Bettiol, Eli Ristevski

**Affiliations:** ^1^ School of Rural Health, Monash University Bendigo Victoria Australia; ^2^ West Gippsland Healthcare Group Warragul Victoria Australia; ^3^ School of Rural Health, Monash University Warragul Victoria Australia

**Keywords:** Australia, cancer, drug utilization, oral anti‐cancer medications, pharmacoepidemiology, time trends

## Abstract

**Purpose:**

Oral anti‐cancer medications (OAMs) are easily administered yet high‐risk treatments. Few studies have investigated national and subnational trends in OAM dispensing. We aimed to examine 10‐year trends in Australia's OAM dispensing at the national level as well as by state/territory and medication type/class.

**Methods:**

Aggregate data on Australia's OAM dispensing and population for 2014–2023 were sourced from Services Australia and the Australian Bureau of Statistics, respectively. Annual OAM dispensing rates (counts per 100 000 population) were calculated overall as well as by state/territory and medication type/class. Percentage change (Δ) in dispensing rates from 2014 to 2023 was determined. Where valid, Mann‐Kendall trend tests were performed.

**Results:**

Australia‐wide from 2014 to 2023, dispensing counts per 100 000 population for any OAMs increased nonlinearly from 3 475 to 3 930 (+Δ13%), hormonal OAMs decreased nonlinearly from 2 659 to 2 225 (−Δ16%), and non‐hormonal OAMs exhibited a significant (*p* < 0.05) near‐linear upward trend from 816 to 1 705 (+Δ109%). This coincided with a significant upward trend in the number of unique non‐hormonal OAMs dispensed Australia‐wide (+Δ187%). Percentage changes in non‐hormonal OAM dispensing rates were greatest for protein kinase inhibitor (PKI) dispensing Australia‐wide (+Δ232%), with a significant, near‐linear upward trend from 286 to 950, and non‐hormonal OAM dispensing in South Australia (+Δ141%), with a significant, near‐linear upward trend from 820 to 1972.

**Conclusions:**

Australia's non‐hormonal OAM dispensing increased over 2014–2023, mostly for PKIs. This likely reflects rising availability of and prescriber/patient demand for these medications, suggesting scope to pilot and expand OAM adherence and safety initiatives.

**Summary**

- Australia‐wide from 2014 to 2023, there was a statistically significant upward trend in the rate of non‐hormonal oral anti‐cancer medication (OAM) dispensing (+Δ109%).
- The significant upward trend in the non‐hormonal OAM dispensing rate Australia‐wide coincided with a statistically significant upward trend in the number of unique non‐hormonal OAMs dispensed Australia‐wide (+Δ187%), suggesting that rising market availability may partly explain the increased dispensing rate.
- The medication class and state/territory with the greatest percentage changes (increases) in non‐hormonal OAM dispensing Australia‐wide over 2014–2023 were protein kinase inhibitors (+Δ232%) and South Australia (+Δ141%), respectively.
- Our results suggest a growing scope for OAM adherence and safety initiatives.

## 1 Introduction

Oral anti‐cancer medications (OAMs) are anti‐cancer medications (AMs) administered in oral dosage forms, including tablets and capsules [1]. Much like parenteral anti‐cancer medications (PAMs), OAMs have been found to improve survival following the diagnosis of particular cancer types [2, 3]. The overarching benefit of OAMs over PAMs is ease of oral administration. Patients taking OAMs have greater autonomy and are able to conveniently self‐administer treatment at home, without needing to travel to cancer centers for relatively invasive parenteral treatment [4]. There is some evidence to suggest that adverse events are somewhat less common with OAMs than with PAMs; however, OAMs are still high‐risk medications in that their adverse events are relatively difficult to detect and severe [1, 4, 5, 6, 7]. While patients taking PAMs have regular contact with clinicians at cancer centers, those taking OAMs typically have reduced clinical contact and, thus, less medication‐related education, support, and monitoring [4, 8]. Non‐hormonal OAM errors and serious adverse events have been reported, for example, at United States (US) cancer centers [9] and in Australian outpatient/community settings [10]. OAM initiation has triggered unplanned emergency department visits and hospitalisations in, for example, 13% and 11% (respectively) of OAM patients in a US setting [11].

Few known studies have examined trends in population‐level dispensing of OAMs. In Manitoba, Canada, counts of patients dispensed OAMs per 100 000 population over the fiscal years 2003/04–2015/16 increased from 222 to 329 for any OAMs, with upward trends from 3 to 34 for targeted OAMs (e.g., the protein kinase inhibitor [PKI] imatinib) and from 154 to 231 for hormonal OAMs (e.g., the aromatase inhibitor [AI] anastrozole), but a downward trend from 37 to 33 for traditional OAMs (e.g., the antimetabolite capecitabine) [12]. Among 37 348 US patients dispensed new targeted OAMs over 2011–2018, 3% were dispensed these medications in 2011 and 19% in 2018 [13]. Upward trends in OAM dispensing have also been found in European countries, including the Netherlands over 2000–2008 [14] and Portugal over 2008–2020 [15]. Nationwide in France over 2006–2014, percentages of people dispensed any OAMs remained stable at 1%; however, there was an upward trend from 1% to 6% for targeted OAMs [16].

Little is known about nationwide OAM dispensing in Australia. While North American and European studies have investigated OAM dispensing over periods of at least 8 years [12, 13, 14, 15, 16], no such long‐term studies have been conducted in Australia. In a study investigating changes in AM dispensing from before to during the COVID‐19 pandemic among a 10% random sample of Australians, there was an upward trend in the non‐hormonal OAM dispensing rate per 100 000 people from approximately 75 in 2017 to approximately 100 in 2020 [17]. This past Australian study did not examine subnational dispensing. As Australia is a large country with a low population density and some state/territory populations that are considerably older, more geographically isolated, and more socioeconomically disadvantaged than others, there is value in also examining Australia's OAM dispensing at the subnational level [18, 19, 20]. No known studies have examined long‐term trends in OAM dispensing at national and subnational population levels in Australia. Examining such trends would highlight the extent to which these medications have been seen in Australia's national and state/territory health systems over time, thereby helping to fill a literature gap while informing models of care intended to optimize OAM access, adherence, and safety.

We aimed to examine trends in OAM dispensing per unit population at the national level in Australia, as well as by Australian state/territory and medication type/class, over 2014–2023. We also aimed to determine, for OAMs, the percentage share of the AM dispensing market Australia‐wide over 2014–2023.

## 2 Methods

### 2.1 Study Design

We undertook an ecological study involving secondary analysis of aggregate, population‐level quantitative data.

### 2.2 Setting

Our study is set in Australia over the 10‐year period 2014–2023. In Australia, medications approved for particular indications by the Therapeutic Goods Administration (TGA) can be sold to the general public. Most TGA‐approved prescription medications dispensed to the general public, including AMs, are subsidized by the Australian Government through the Pharmaceutical Benefits Scheme (PBS) and/or the Repatriation Pharmaceutical Benefits Scheme (RPBS) [21]. Some TGA‐approved prescription medications are not available through the PBS or RPBS and, thus, can only be accessed through other, smaller‐scale initiatives or at higher costs. The general public can opt to access those medications that are listed on the PBS or RPBS schedules outside of the PBS and RPBS, although this is generally unlikely for AMs due to the relatively strong financial disincentive.

### 2.3 Data Source

We sourced Australia‐wide OAM dispensing data for each year of the period 2014–2023. Aggregate data on dispensing counts for individual OAM products and the group of any (oral/parenteral) AMs subsidized under the PBS or RPBS, stratified by state/territory of pharmacy and year of claim processing, were sourced from Services Australia's Pharmaceutical Benefits Schedule Item Reports [22] and Pharmaceutical Benefits Schedule Group Reports [23], respectively. Year of dispensing claim processing acted as a marker of year of actual dispensing. Additionally, aggregate data on Australia's mid‐year estimated resident population, stratified by residential state/territory and year, were sourced from the Australian Bureau of Statistics (ABS) [24]. All data were downloaded in Microsoft Excel files.

### 2.4 Definition of Oral Anti‐Cancer Medications

OAMs were defined as PBS medication products corresponding to Anatomical Therapeutic Chemical (ATC) Index level 2 codes L01 (Antineoplastic agents), L02 (Endocrine therapy) or L04 (Immunosuppressants) except those that were unavailable in an oral dosage form in Australia, not indicated for cancer in Australia, and/or neither PBS nor RPBS subsidized [1, 25, 26]. Cyclophosphamide, mercaptopurine, and methotrexate were excluded because they are mostly used for autoimmune diseases [1, 17] and reasons for prescribing/dispensing (i.e., individuals' conditions for which medications were prescribed/dispensed) were unavailable in our data sources. Exclusion of these medications is consistent with past OAM studies that lacked data on reasons for prescribing/dispensing [14, 17]. Overall, 77 different OAMs were included in our study (Appendix A, Table A1).

### 2.5 Oral Anti‐Cancer Medication Types and Classes

OAM types were assigned using medication classes corresponding to ATC level 3 codes [26]: hormonal OAMs (hormonal agents [L02B]), non‐hormonal OAMs (alkylating agents [L01A], antimetabolites [L01B], natural products [L01C], PKIs [L01E], other antineoplastic agents [L01X], and immunosuppressants [L04A]), and any (hormonal and non‐hormonal combined) OAMs.

### 2.6 Definition of Any (Oral/Parenteral) Anti‐Cancer Medications

Any (oral/parenteral) AMs were defined similarly to OAMs (Section 2.4), but without the exclusion of parenteral products.

### 2.7 Data Analysis

For each of any, hormonal, and non‐hormonal OAMs (medication types), dispensing counts were calculated by year and by state/territory within each year. Dispensing counts for non‐hormonal OAMs were also calculated by medication classes, while dispensing counts for each of any, hormonal, and non‐hormonal OAMs were calculated by individual medications.

OAM dispensing counts were divided by corresponding population estimates and multiplied by 100 000 to give dispensing rates per 100 000 population. OAM dispensing rates per 100 000 population were further analyzed by calculating percentage change (Δ) from 2014 to 2023. Where valid (i.e., when rates only either increased monotonically or decreased monotonically over time), Mann‐Kendall trend tests were performed. *p*‐values < 0.05 denoted statistical significance.

To assess OAM market share, annual Australia‐wide dispensing counts for any OAMs, hormonal OAMs, and non‐hormonal OAMs were re‐expressed as percentages of dispensing counts among any (oral/parenteral) AMs.

Data were analyzed and visualized using Microsoft Excel 2019 (Microsoft Corporation, Redmond, WA, USA) and Stata Version 15.0 (StataCorp, College Station, Texas, USA).

### 2.8 Ethical Considerations

Ethical approval to undertake this study has been granted by the Monash University Human Research Ethics Committee (ID: 42043). Informed consent was not required due to the use of routinely collected, publicly available aggregate data.

## 3 Results

**FIGURE 1:**
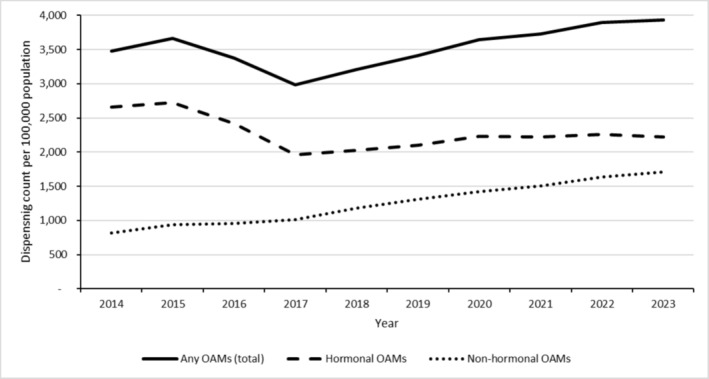
Dispensing rates (counts per 100 000 population) for any (total), hormonal, and non‐hormonal oral anti‐cancer medications, by year, Australia, 2014–2023. OAM, oral anti‐cancer medication.

Australia‐wide from 2014 to 2023, annual dispensing counts per 100 000 population for any OAMs increased nonlinearly and non‐monotonically from 3 475 to 3 930 (+Δ13%), hormonal OAMs decreased nonlinearly and non‐monotonically from 2 659 to 2 225 (−Δ16%), and non‐hormonal OAMs increased near‐linearly and monotonically from 816 to 1 705 (+Δ109%) (Figure 1). The nonlinear, non‐monotonic trend in hormonal OAMs involved a slight increase over 2014–2015, a sharp decrease over 2015–2017, and a slight increase over 2017–2023. There was a statistically significant upward trend in the rate of non‐hormonal OAM dispensing Australia‐wide from 2014 to 2023 (*p*‐value < 0.001). This significant upward trend in the non‐hormonal OAM dispensing rate coincided with a significant (*p* < 0.001) upward trend in the number of individual non‐hormonal OAMs dispensed: an increase from 23 different agents in 2014 to 66 different agents in 2023 (+Δ187%) (Appendix A, Table A1).

**FIGURE 2:**
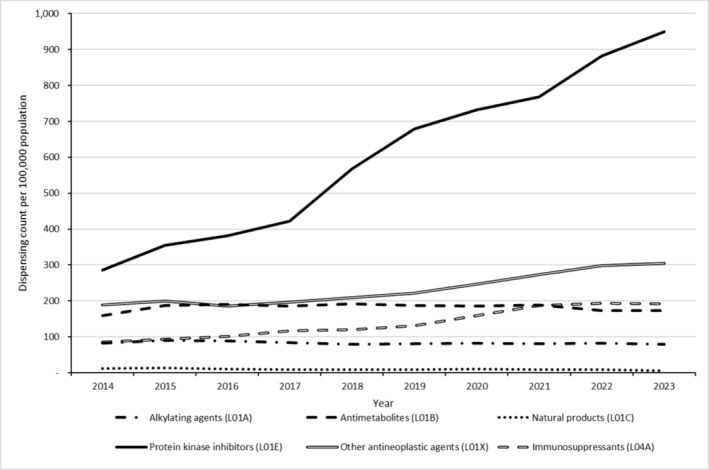
Dispensing rates (counts per 100 000 population) for non‐hormonal oral anticancer medications by class and year, Australia, 2014–2023.

Non‐hormonal OAM dispensing rates over time varied by medication class (Figure 2). Australia‐wide from 2014 to 2023, annual dispensing rates per 100 000 population for PKIs increased near‐linearly and monotonically from 286 to 950 (+Δ232%), immunosuppressants increased near‐linearly and monotonically from 86 to 192 (+Δ124%), other antineoplastic agents increased non‐linearly and non‐monotonically from 189 to 305 (+Δ61%), antimetabolites increased non‐linearly and non‐monotonically from 160 to 173 (+Δ8%), alkylating agents decreased non‐linearly and non‐monotonically from 82 to 79 (−Δ4%), and natural products decreased non‐linearly and non‐monotonically from 12 to 6 (−Δ50%). There were statistically significant upward trends in rates of PKI and immunosuppressant dispensing Australia‐wide from 2014 to 2023 (*p*‐values < 0.001).

Dispensing rates over time and percentage changes for each of the 77 individual OAMs are shown in Appendix A (Table A1). Most (56%) of the 77 individual OAMs dispensed over the 10‐year period 2014–2023 were PKIs. Upward trends in dispensing rates over time were observed for 31 of 43 PKIs (highest: +Δ7 223% for lenvatinib) and for individual OAMs in other classes. The individual hormonal OAMs anastrozole and letrozole exhibited a similar year‐on‐year trend to the overall hormonal OAM trend: an increase over 2014–2015 followed by a sharp decrease over 2015–2017 and then an increase over 2017–2023.

**FIGURE 3:**
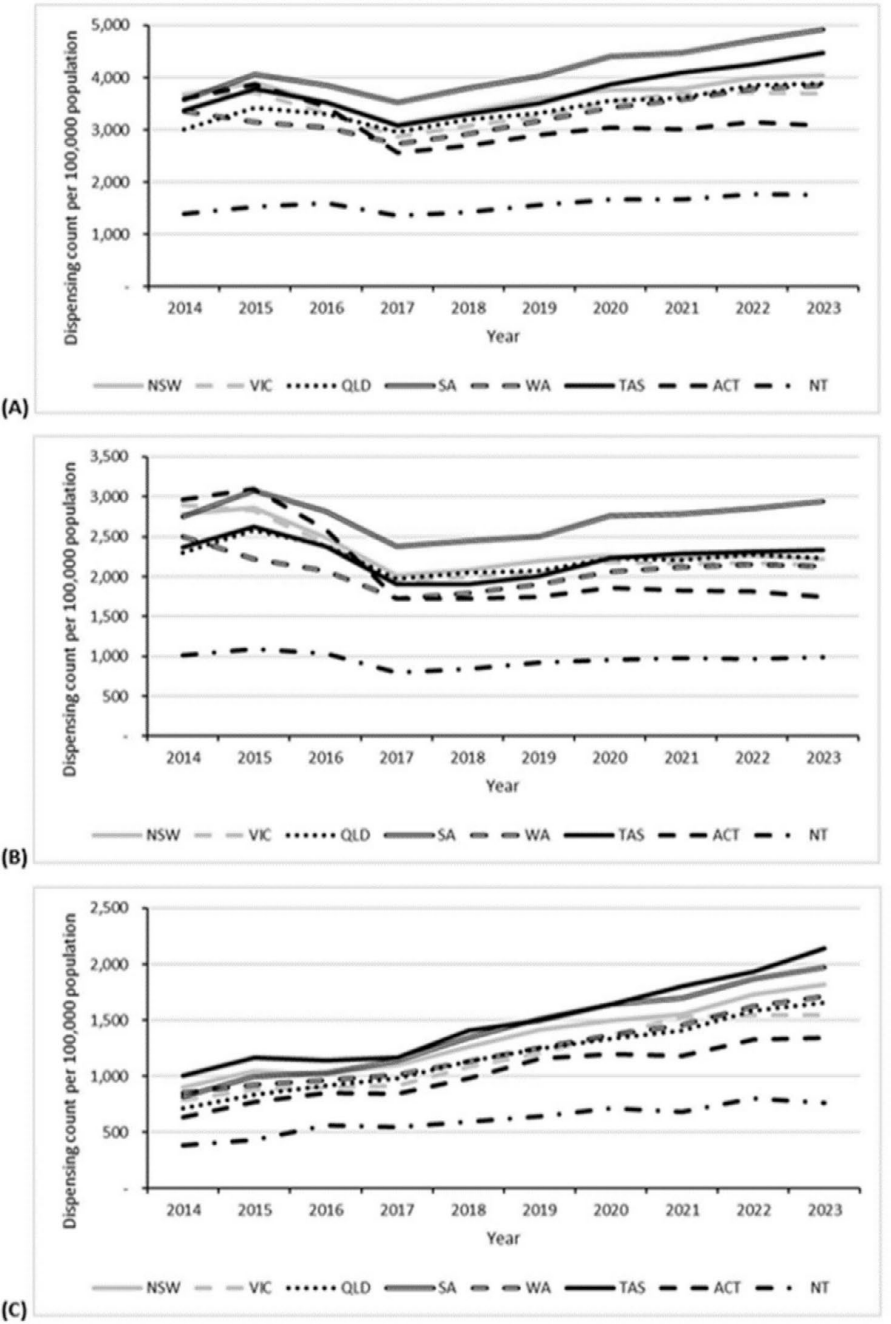
Dispensing rates (counts per 100 000 population) for (A) any (total), (B) hormonal, and (C) non‐hormonal oral anti‐cancer medications, by state/territory and year, Australia, 2014–2023. ACT, Australian Capital Territory; NSW, New South Wales; NT, Northern Territory; QLD, Queensland; SA, South Australia; TAS, Tasmania; VIC, Victoria; WA, Western Australia.

Dispensing rates for each of any, hormonal, and non‐hormonal OAMs varied by the state/territory of the dispensing pharmacy (Figure 3). During most years, rates of dispensing of any and hormonal OAMs were highest for South Australia (SA) while rates of non‐hormonal OAM dispensing were highest for Tasmania. Across the entire 10‐year period, dispensing rates for any, hormonal, and non‐hormonal OAMs were markedly lower in the Northern territory (NT) than in any other Australian state/territory. SA had the highest percentage change in any (+Δ37%), hormonal (+Δ7%), and non‐hormonal (+Δ141%) OAM dispensing rates over 2014–2023. Statistically significant upward trends in non‐hormonal OAM dispensing rates were observed for each of Australia's eight states/territories (*p*‐values < 0.001).

**FIGURE 4:**
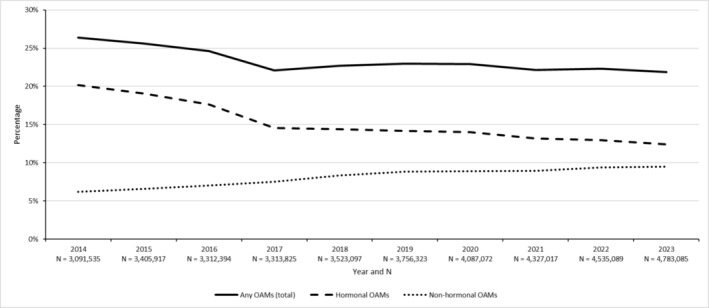
Dispensing counts for any (total), hormonal, and non‐hormonal oral anti‐cancer medications, expressed as percentages of all (parenteral/oral) anti‐cancer medications, by year, Australia, 2014–2023. *N*—Sample size for the denominator (dispensing count for all [parenteral/oral] anti‐cancer medications), OAM, oral anti‐cancer medications.

Regarding market share, the percentage of AM dispensing events that were for OAMs decreased non‐linearly and non‐monotonically from 26% in 2014 to 22% in 2023 (−Δ17%), hormonal OAMs decreased non‐linearly and non‐monotonically from 20% in 2014 to 12% in 2023 (−Δ39%), and non‐hormonal OAMs increased near‐linearly and monotonically from 6% in 2014 to 10% in 2023 (+Δ53%) (Figure 4). The upward trend in non‐hormonal OAM dispensing was statistically significant in the market‐share analysis (*p* < 0.001).

## 4 Discussion

Consistent with past Australian [17] and international [12, 14, 15] studies, our study found that annual rates of any OAM dispensing Australia‐wide increased over 2014–2023. We observed decreasing hormonal OAM dispensing, yet a significant upward trend in non‐hormonal OAM dispensing. The +Δ109% increase in the non‐hormonal OAM dispensing rate and +Δ187% increase in the number of individual non‐hormonal OAMs dispensed likely reflect rising market availability of, prescriber/patient demand for, and access to these potentially life‐saving and convenient, yet high‐risk, medications. Demand for AMs is expected to increase over time in countries with aging populations, such as Australia, because cancers are primarily diseases of the aged [27, 28] and cancer survivorship is becoming increasingly common [29]. Medical oncologists and hematologists may be increasingly prescribing non‐hormonal OAMs due to ever‐rising availability and because they perceive that the benefits of oral administration (e.g., patient preference for greater convenience and autonomy) outweigh the corresponding challenges (e.g., primary and secondary non‐adherence, and adverse events) [4]. Despite the increased dispensing of non‐hormonal OAMs Australia‐wide, however, the level of clinical supervision and support available to people taking these medications still tends to be lower than for those on PAMs [8].

The observed trend in the hormonal OAM dispensing rate (a rise over 2014–2015, a sharp decline over 2015–2017, and an increase from 2017) was also observed for two AIs indicated for breast cancer: anastrozole and letrozole. This same trend in anastrozole and letrozole dispensing was observed nationwide in the US [30]. The authors of this US study suggested reasons for the sharp decline in AI dispensing over 2015–2017, including an evidence‐based shift from longer to shorter periods of extended AI therapy around this time [30]. Our study's decline in anastrozole, letrozole, and overall hormonal OAM dispensing rates could reflect a shift to fewer years of anastrozole and letrozole therapy among Australian breast cancer survivors. Future studies could investigate long‐term trends in durations of hormonal OAM dispensing among breast cancer survivors using patient‐level data.

Australia‐wide, the non‐hormonal OAM medication class with the greatest percentage change (increase) in dispensing was the PKI class (+Δ232%). PKI dispensing drove the overall trend observed for non‐hormonal OAMs. This finding is consistent with a past Portuguese study, which found that the OAM class with the greatest percentage increase in dispensing over 13 years was the PKI class [15]. PKIs are important 21st‐century medications that target different members of the protein kinase family. In our Australian study, lenvatinib was the PKI with the most rapid increase in dispensing. Lenvatinib has approved indications for endometrial, renal, liver, and thyroid cancers, and can lead to serious adverse effects (e.g., renal, liver, and heart failure) [1]. Approximately 80 PKIs have been approved by the US Food and Drug Administration, including seven in 2023 [31]. Of them, 46 have been marketed in Australia [1] and 43 were dispensed in our study.

The Australian state/territory with the greatest percentage change (increase) in the non‐hormonal OAM dispensing rate was the mainland state of SA (+Δ141%). Given SA had only the fifth highest OAM dispensing counts (as opposed to rates) and population estimates among Australia's eight states/territories (data not shown but available online [22, 24]), it is unlikely that increased dispensing in SA drove the trend in non‐hormonal OAM dispensing Australia‐wide. The finding for SA appears to be unrelated to cancer incidence. Over 2014–2020, SA had the lowest percentage change in crude cancer incidence of any Australian state/territory (+Δ8.89%) and Australia's third lowest percentage change in age‐standardized cancer incidence rate (−Δ4.13%) (author calculations) [27]. While SA had the greatest percentage increase in non‐hormonal OAM dispensing rates over 2014–2023, the island state of Tasmania had the highest non‐hormonal OAM dispensing rates during most years of the study period. This may partly reflect the fact that Tasmania has the oldest population of any Australian state/territory (median age = 42 years in 2022) [18]. Tasmania's high non‐hormonal OAM dispensing rates are noteworthy because, unlike most Australian states/territories, Tasmania lacks a metropolitan center [19]. Tasmanians who take non‐hormonal OAMs may receive even less education, support, and monitoring than people taking these medications in most of Australia's mainland states/territories [32]. Conversely, NT had the lowest OAM dispensing rates during every year over 2014–2023. This may partly reflect lower need for OAMs, as NT has Australia's youngest population (median age = 33.5 years in 2022) [18], and/or lower financial and geographic accessibility of OAMs, as NT is a severely socioeconomically disadvantaged and entirely non‐metropolitan territory [19, 20]. Prescriber/patient preferences and other accessibility considerations may also explain state/territory‐level variation in dispensing across Australia; future investigation is warranted.

Our study has presented data on OAM dispensing rates for Australia's highest‐level subnational areas: the level of states/territories. As population age, cancer incidence/prevalence, socioeconomic status, and health service accessibility vary across smaller geographic areas within each state/territory, however, there is a need to explore data on Australia's OAM dispensing rates for smaller geographic areas (e.g., Statistical Area Level 3 areas) [33] as well as remoteness areas [34]. This is another avenue for future research.

The present study's strengths include its relatively long and up‐to‐date study period of 2014–2023, and the analysis of nationwide population‐level data from Services Australia's Pharmaceutical Benefits Schedule Item and Group Reports [22, 23]. These reports are novel, up‐to‐date (monthly), and publicly available sources of Australian administrative data for a study of OAM dispensing trends, contrasting with the dataset of dispensing records for a 10% sample of Australians that is only available on request [17, 35]. This study does, however, also have limitations. State/territory of pharmacy may differ from patients' residential state/territory, while claim processing year may differ from supply year. Additionally, as only PBS‐ or RPBS‐subsidized dispensing was captured, OAMs and PAMs provided off‐label, via private dispensing, or through compassionate access were excluded from our study. Those AMs with similar private and PBS‐ or RPBS‐subsidized dispensing costs (e.g., the hormonal OAM tamoxifen) may be more likely to be dispensed outside the PBS and RPBS than those AMs for which private dispensing costs are substantially higher. At the time of writing, 22 AMs are only available in Australia without PBS/RPBS subsidization: 14 PAMs from a wide range of classes and 8 OAMs from the alkylating agent (lomustine and procarbazine), PKI (regorafenib, salumetinib and vandetanib), and other antineoplastics (anagrelide, sotorasib and tretinoin) classes [1]. Our lack of data on AMs supplied outside the PBS and RPBS means that OAM dispensing rates per 100 000 population were likely underestimated to an extent and that the OAM market share could have been either underestimated or overestimated. Lastly, OAM dispensing rates were not age‐standardized or restricted to higher‐risk older ages (e.g., 18+ years) because our data sources lacked age‐specific dispensing data [22, 23], although we did partially address this limitation in the market‐share analysis with a cancer‐specific denominator. Including younger ages likely led to slightly underestimated dispensing rates.

In conclusion, Australia's non‐hormonal OAM dispensing rates increased over 2014–2023, mostly for PKIs and in SA. There is rising availability of, prescriber/patient demand for, and access to these potentially life‐saving yet high‐risk medications in all Australian states and territories, suggesting a growing scope to expand OAM adherence and safety initiatives across metropolitan and non‐metropolitan areas. Hospitals, general practices, and community pharmacies could pilot and expand innovative models of oral anti‐cancer care that are tailored to particular geographic locations and aimed at improving non‐hormonal OAM education, support, and monitoring, particularly in relation to PKIs. Such care models could take the form of clinics involving shared care among health professionals from multiple disciplines and practice settings, with leadership by one or more health professionals (e.g., a nurse‐ and/or pharmacist‐led clinic) [36, 37, 38].

### 4.1 Plain Language Summary

Oral anti‐cancer medications are potentially life‐saving treatments for cancer that are easily and conveniently administered by mouth in oral dosage forms, including tablets and capsules. There is a high risk that these medications will cause harm. Despite the benefits and risks of oral anti‐cancer medications, little is known about how the dispensing (i.e., supply) of these medications has changed over time. We examined how oral anti‐cancer medication dispensing changed Australia‐wide and in Australian states and territories over 10 years. Data on Australia's oral anti‐cancer medication dispensing and population for 2014–2023 were obtained from Services Australia and the Australian Bureau of Statistics, respectively. Annual oral anti‐cancer medication dispensing rates (counts per 100 000 population) were calculated overall and by state/territory and medication type/class. Statistical tests for time trends were performed. Australia‐wide from 2014 to 2023, there was a statistically significant upward trend in the non‐hormonal oral anti‐cancer medication dispensing rate. This coincided with a statistically significant upward trend in the number of unique non‐hormonal oral anti‐cancer medications dispensed, suggesting that rising market availability may partly explain the increased dispensing rate. The medication class and state/territory with the greatest percentage increases in non‐hormonal oral anti‐cancer medication dispensing Australia‐wide over 2014–2023 were protein kinase inhibitors and South Australia, respectively. Our results suggest a growing scope for oral anti‐cancer medication use and safety initiatives.

## Consent

The authors have nothing to report.

## Conflicts of Interest

The authors declare no conflicts of interest.

## Appendix A Individual Oral Anti‐Cancer Medications

**TABLE A1:** Dispensing rate (count per 100 000 population), and percentage change, for each individual (unique/different) oral anti‐cancer medication by class and year, Australia, 2014–2023.

		**Dispensing rate (count per 100000 population)** *****										**% change**
**Individual OAM**	**ATC class**	**2014**	**2015**	**2016**	**2017**	**2018**	**2019**	**2020**	**2021**	**2022**	**2023**	**from earliest year to 2023**
Busulfan	Alkylating agents (L01A)	1.44	1.77	1.53	1.41	1.33	1.11	1.19	1.13	0.95	0.74	–48.75%
Chlorambucil	Alkylating agents (L01A)	15.04	13.84	11.74	12.04	10.40	9.07	9.22	6.75	4.81	3.11	–79.33%
Melphalan	Alkylating agents (L01A)	9.66	8.05	5.03	3.23	2.54	2.48	1.88	1.31	1.28	1.10	–88.65%
Temozolomide	Alkylating agents (L01A)	56.19	67.32	70.58	67.44	65.25	68.43	70.81	71.54	75.59	74.21	+32.07%
Azacitidine	Antimetabolites (L01B)	—	—	—	—	—	—	—	—	—	0.21	N/A
Capecitabine	Antimetabolites (L01B)	151.75	178.64	181.77	176.37	178.23	162.56	164.20	166.92	152.52	145.37	–4.20%
Decitabine‐Cedazuridine	Antimetabolites (L01B)	—	—	—	—	—	—	—	—	0.34	5.39	+1 492.47%
Fludarabine	Antimetabolites (L01B)	3.41	3.31	3.23	3.23	6.47	4.75	1.21	0.95	0.65	0.63	–81.49%
Tioguanine	Antimetabolites (L01B)	4.55	5.24	5.46	6.21	6.51	6.75	8.04	9.59	10.91	10.59	+132.48%
Trifluridine‐tipiracil	Antimetabolites (L01B)	—	—	—	—	0.54	12.99	12.77	11.92	9.33	10.88	+1 896.18%
Etoposide	Natural products (L01C)	6.21	6.54	6.29	5.30	5.38	5.90	7.13	6.73	5.99	4.40	–29.15%
Vinorelbine	Natural products (L01C)	6.06	6.94	4.69	4.03	3.51	3.04	3.06	2.65	2.51	1.72	–71.64%
Abemaciclib	PKIs (L01E)	—	—	—	—	—	—	3.03	8.82	21.79	32.34	+966.20%
Acalabrutinib	PKIs (L01E)	—	—	—	—	—	—	0.92	8.60	19.77	26.96	+2 830.61%
Afatinib	PKIs (L01E)	—	—	—	—	1.22	4.99	5.62	3.53	2.65	2.12	+74.17%
Alectinib	PKIs (L01E)	—	—	—	—	6.17	9.24	12.06	14.20	15.06	15.18	+146.30%
Asciminib	PKIs (L01E)	—	—	—	—	—	—	—	—	—	4.62	N/A
Axitinib	PKIs (L01E)	—	0.12	5.10	7.41	4.47	3.11	3.04	2.61	3.29	3.49	+2 767.10%
Binimetinib	PKIs (L01E)	—	—	—	—	—	—	3.52	8.71	9.69	11.08	+214.42%
Brigatinib	PKIs (L01E)	—	—	—	—	—	—	0.32	0.74	1.10	1.29	+297.91%
Cabozantinib	PKIs (L01E)	—	—	—	—	2.34	7.20	8.77	11.19	15.43	17.11	+631.23%
Ceritinib	PKIs (L01E)	—	—	—	1.50	0.78	0.36	0.29	0.21	0.08	0.08	–94.73%
Cobimetinib	PKIs (L01E)	—	—	—	0.88	2.28	1.75	1.77	1.51	0.79	0.59	–32.78%
Crizotinib	PKIs (L01E)	—	1.66	5.05	5.11	3.14	2.46	2.48	2.20	1.59	1.28	–23.04%
Dabrafenib	PKIs (L01E)	14.67	23.28	23.94	23.63	23.71	22.32	22.30	17.81	15.20	13.77	–6.14%
Dasatinib	PKIs (L01E)	27.50	35.08	36.25	38.98	41.97	46.05	50.22	52.33	52.71	52.83	+92.15%
Encorafenib	PKIs (L01E)	—	—	—	—	—	—	3.56	8.76	14.17	15.96	+348.81%
Entrectinib	PKIs (L01E)	—	—	—	—	—	—	0.05	0.74	1.37	1.85	+3 283.77%
Erlotinib	PKIs (L01E)	19.10	25.50	23.90	26.56	26.79	24.99	24.70	12.21	5.74	3.39	–82.27%
Everolimus	PKIs (L01E)	37.57	60.24	54.19	57.98	64.45	71.66	73.28	73.18	81.01	75.01	+99.65%
Gefitinib	PKIs (L01E)	7.66	9.89	9.23	10.52	11.83	11.85	12.62	6.58	3.69	2.59	–66.13%
Gilteritinib	PKIs (L01E)	—	—	—	—	—	—	—	—	0.19	0.76	+304.58%
Ibrutinib	PKIs (L01E)	—	—	—	1.59	43.35	57.54	63.00	59.89	51.00	41.09	+2 484.71%
Idelalisib	PKIs (L01E)	—	—	—	1.59	41.15	49.67	53.20	49.69	42.53	35.11	+2 108.59%
Imatinib	PKIs (L01E)	115.42	120.31	110.50	105.67	103.24	108.10	111.45	108.76	108.19	94.26	–18.34%
Lapatinib	PKIs (L01E)	5.28	2.69	1.35	1.72	1.75	1.78	1.69	1.81	1.45	1.11	–79.02%
Larotrectinib	PKIs (L01E)	—	—	—	—	—	—	—	—	0.04	0.25	+554.30%
Lenvatinib	PKIs (L01E)	—	—	0.24	4.67	5.60	10.34	14.77	12.65	11.84	17.86	+7 223.42%
Lorlatinib	PKIs (L01E)	—	—	—	—	—	—	0.55	2.36	2.91	4.46	+717.75%
Midostaurin	PKIs (L01E)	—	—	—	—	0.02	1.44	1.70	1.86	1.36	1.39	+5 678.85%
Nilotinib	PKIs (L01E)	22.37	27.24	27.97	28.81	29.77	31.07	31.19	30.89	31.37	28.58	+27.73%
Nintedanib	PKIs (L01E)	—	—	—	8.54	27.72	38.88	47.41	54.15	66.12	92.04	+978.37%
Osimertinib	PKIs (L01E)	—	—	—	—	—	10.63	14.52	46.68	64.00	71.67	+573.97%
Palbociclib	PKIs (L01E)	—	—	—	—	—	—	—	—	66.83	79.02	+18.24%
Pazopanib	PKIs (L01E)	8.79	10.03	10.38	10.52	13.62	15.35	14.93	14.53	12.11	9.78	+11.23%
Ponatinib	PKIs (L01E)	—	0.07	1.11	1.36	1.25	1.80	2.00	2.38	2.53	2.77	+4 023.77%
Ribociclib	PKIs (L01E)	—	—	—	—	19.94	58.23	56.00	58.94	63.24	71.22	+257.13%
Ripretinib	PKIs (L01E)	—	—	—	—	—	—	—	0.05	1.47	1.40	+2 468.96%
Ruxolitinib	PKIs (L01E)	—	—	25.17	37.48	44.12	47.76	53.78	57.36	60.12	72.13	+186.61%
Sorafenib	PKIs (L01E)	8.64	12.00	8.94	8.92	9.05	6.03	3.72	1.97	1.24	0.77	–91.14%
Sunitinib	PKIs (L01E)	19.38	18.93	14.56	13.32	11.42	10.16	9.61	10.50	8.17	6.53	–66.29%
Tepotinib	PKIs (L01E)	—	—	—	—	—	—	—	—	0.11	1.80	+1 570.63%
Trametinib	PKIs (L01E)	—	7.07	23.53	24.18	24.08	22.60	22.70	18.19	15.41	13.92	+96.91%
Vemurafenib	PKIs (L01E)	—	—	—	1.05	2.68	2.02	2.09	1.69	1.01	0.82	–21.94%
Zanubrutinib	PKIs (L01E)	—	—	—	—	—	—	—	—	3.48	19.74	+467.49%
Hydroxycarbamide (Hydroxyurea)	Other antineoplastics (L01X)	189.20	199.97	185.97	195.71	205.15	209.77	223.11	225.35	233.24	230.52	+21.84%
Niraparib	Other antineoplastics (L01X)	—	—	—	—	—	—	—	—	0.03	0.74	+2 048.45%
Olaparib	Other antineoplastics (L01X)	—	—	—	—	0.06	4.81	6.76	15.83	19.66	20.47	+36 400.45%
Selinexor	Other antineoplastics (L01X)	—	—	—	—	—	—	—	—	0.31	1.43	+360.56%
Sonidegib	Other antineoplastics (L01X)	—	—	—	—	0.40	1.06	1.55	1.78	2.14	2.12	+428.53%
Venetoclax	Other antineoplastics (L01X)	—	—	—	—	—	3.43	12.25	26.84	40.31	46.18	+1 247.92%
Vismodegib	Other antineoplastics (L01X)	—	—	—	0.90	2.44	2.12	2.73	2.90	2.64	2.98	+231.26%
Vorinostat	Other antineoplastics (L01X)	—	—	—	0.29	0.83	0.64	0.46	0.57	0.63	0.50	+69.25%
Abiraterone	Hormonal agents (L02B)	52.22	53.61	44.64	46.31	52.89	58.98	63.39	63.48	70.00	68.72	+31.61%
Anastrozole	Hormonal agents (L02B)	994.03	1051.23	900.15	621.81	619.69	619.87	638.77	618.01	612.33	577.67	–41.89%
Apalutamide	Hormonal agents (L02B)	—	—	—	—	—	—	—	—	—	10.21	N/A
Bicalutamide	Hormonal agents (L02B)	195.20	206.78	187.71	188.96	179.15	168.29	161.97	152.46	143.21	122.91	–37.03%
Darolutamide	Hormonal agents (L02B)	—	—	—	—	—	—	—	1.10	19.65	30.59	+2 686.66%
Enzalutamide	Hormonal agents (L02B)	0.01	47.25	72.79	88.86	102.84	111.90	122.87	123.91	128.91	150.83	+1 770 278.50%
Exemestane	Hormonal agents (L02B)	15.48	93.69	106.30	133.70	151.91	163.04	172.98	175.67	172.41	170.10	+998.54%
Flutamide	Hormonal agents (L02B)	3.04	2.49	1.91	0.49	0.65	0.21	0.19	0.14	0.11	0.07	–97.78%
Letrozole	Hormonal agents (L02B)	767.53	822.94	740.64	527.63	564.02	617.95	690.04	722.37	757.82	769.40	+0.24%
Tamoxifen	Hormonal agents (L02B)	625.25	438.72	354.66	351.59	351.76	355.76	375.66	357.80	345.96	320.58	–48.73%
Toremifene	Hormonal agents (L02B)	6.00	6.10	4.99	4.73	4.31	3.73	3.86	3.63	3.55	3.57	–40.48%
Lenalidomide	Immunosuppressants (L04A)	43.70	51.15	57.82	72.76	88.74	100.55	133.31	165.79	170.07	170.54	+290.21%
Pomalidomide	Immunosuppressants (L04A)	—	1.61	8.03	9.12	7.06	11.81	15.49	17.39	20.47	19.52	+1 110.68%
Thalidomide	Immunosuppressants (L04A)	42.21	40.75	35.94	35.35	24.74	18.11	9.75	4.40	2.83	2.05	–95.13%
No. of individual OAMs	All OAMs	32	37	39	47	55	57	64	66	74	77	140.63%
No. of individual hormonal OAMs	All hormonal OAMs	9	9	9	9	9	9	9	10	10	11	22.22%
No. of individual non‐hormonal OAMs	All non‐hormonal OAMs	23	28	30	38	46	48	55	56	64	66	186.96%

Abbreviations: ATC, Anatomical Therapeutic Chemical; OAM, oral anti‐cancer medication; PKI, protein kinase inhibitor.

* Unless otherwise stated (for “No. of individual OAMs”, “No. of individual hormonal OAMs” and “No. of individual non‐hormonal OAMs)”.

## References

[1] Australian Medicines Handbook Pty Ltd , “Australian Medicines Handbook,” accessed April 19, 2024, https://amhonline.amh.net.au/.

[2] C. Twelves , A. Wong , M. P. Nowacki , et al., “Capecitabine as Adjuvant Treatment for Stage III Colon Cancer,” New England Journal of Medicine 352, no. 26 (2005): 2696–2704, 10.1056/NEJMoa043116. 15987918

[3] C. Zhu , X. Ma , and Y. Hu , “Safety and Efficacy Profile of Lenvatinib in Cancer Therapy: A Systematic Review and Meta‐Analysis,” Oncotarget 7, no. 28 (2016): 44545–44557, 10.18632/oncotarget.10019. 27329593 PMC5190117

[4] T. Halfdanarson and A. Jatoi , “Oral Cancer Chemotherapy: The Critical Interplay Between Patient Education and Patient Safety,” Current Oncology Reports 12, no. 4 (2010): 247–252, 10.1007/s11912-010-0103-6. 20437116 PMC9651012

[5] A. Bourmaud , C. Pacaut , A. Melis , et al., “Is Oral Chemotherapy Prescription Safe for Patients? A Cross‐Sectional Survey,” Annals of Oncology 25, no. 2 (2014): 500–504, 10.1093/annonc/mdt553. 24406423

[6] S. Weingart , L. Zhang , M. Sweeney , et al., “Chemotherapy Medication Errors,” Lancet Oncology 19, no. 4 (2018): e191–e199, 10.1016/S1470-2045(18)30094-9. 29611527

[7] Australian Commission on Safety and Quality in Health Care , “APINCHS Classification of High Risk Medicines,” accessed August 30, 2024, https://www.safetyandquality.gov.au/our‐work/medication‐safety/high‐risk‐medicines/apinchs‐classification‐high‐risk‐medicines.

[8] J. P. Richmond , M. G. Kelly , A. Johnston , et al., “Current Management of Adults Receiving Oral Anti‐Cancer Medications: A Scoping Review,” European Journal of Oncology Nursing 54 (2021): 102015, 10.1016/j.ejon.2021.102015. 34500319

[9] S. Weingart , J. Flug , D. Brouillard , et al., “Oral Chemotherapy Safety Practices at US Cancer Centres: Questionnaire Survey,” British Medical Journal 334, no. 7590 (2007): 407, 10.1136/bmj.39069.489757.55. 17223629 PMC1804126

[10] State Government of Victoria , “Caution With Oral Chemotherapy for Cancer,” accessed August 22, 2024, https://www.safetyandquality.gov.au/sites/default/files/migrated/Oral‐for‐health‐services‐Victorian‐Department‐of‐Health.pdf.

[11] A. Sikorskii , C. W. Given , S. Chang , et al., “Patient Reported Outcomes and Unscheduled Health Services Use During Oral Anti‐Cancer Treatment,” Journal of Pain and Symptom Management 65, no. 2 (2023): e115–e121, 10.1016/j.jpainsymman.2022.10.003. 36244640 PMC9840667

[12] C. Leong , P. Craykowski , M. Geirnaert , et al., “Outpatient Oral Anticancer Agent Utilization and Costs in Manitoba From 2003 to 2016: A Population‐Based Study,” Canadian Journal of Public Health 112, no. 3 (2021): 530–540, 10.17269/s41997-020-00464-6. 33471346 PMC8076393

[13] M. Fu , H. Naci , C. M. Booth , et al., “Real‐World Use of and Spending on New Oral Targeted Cancer Drugs in the US, 2011–2018,” JAMA Internal Medicine 181, no. 12 (2021): 1596–1604, 10.1001/jamainternmed.2021.5983. 34661604 PMC8524355

[14] L. Timmers , J. J. Beckeringh , M. P. P. van Herk‐Sukel , et al., “Use and Costs of Oral Anticancer Agents in the Netherlands in the Period 2000–2008,” Pharmacoepidemiology and Drug Safety 21, no. 10 (2012): 1036–1044, 10.1002/pds.2225. 21956857

[15] A. Moreira , C. Bernardo , C. Ramos , et al., “National Trends in the Use of Oral Chemotherapy Over 13 Years,” Frontiers in Pharmacology 13 (2022): 909948, 10.3389/fphar.2022.909948. 36034797 PMC9399396

[16] P. Bosco‐Levy , P. de Boissieu , A. Gouverneur , et al., “National Trends in Use and Costs of Oral Anticancer Drugs in France: An 8‐Year Population‐Based Study,” Pharmacoepidemiology and Drug Safety 26 (2017): 1233–1241, 10.1002/pds.4282. 28771878

[17] M. Tang , B. Daniels , M. Aslam , et al., “Changes in Systemic Cancer Therapy in Australia During the COVID‐19 Pandemic: A Population‐Based Study,” Lancet Regional Health‐Western Pacific 14 (2021): 100226, 10.1016/j.lanwpc.2021.100226. 34368796 PMC8329989

[18] Australian Bureau of Statistics , “Regional Population by Age and Sex,” accessed August 26, 2024, https://www.abs.gov.au/statistics/people/population/regional‐population‐age‐and‐sex/2022.

[19] Australian Government Department of Health and Aged Care , “Modified Monash Model,” accessed August 28, 2024, https://www.health.gov.au/topics/rural‐health‐workforce/classifications/mmm.

[20] Australian Bureau of Statisitcs , “Socio‐Economic Indexes for Areas (SEIFA), Australia,” accessed 16 January, 2025, https://www.abs.gov.au/statistics/people/people‐and‐communities/socio‐economic‐indexes‐areas‐seifa‐australia/latest‐release.

[21] Australian Government Department of Health and Aged Care , “About the PBS,” accessed November 4, 2024, https://www.pbs.gov.au/info/about‐the‐pbs.

[22] Australian Government Services Australia , “Pharmaceutical Benefits Schedule Item Reports,” accessed January 25, 2024, http://medicarestatistics.humanservices.gov.au/statistics/pbs_item.jsp.

[23] Australian Government Services Australia , “Pharmaceutical Benefits Schedule Group Reports,” accessed January 25, 2024, http://medicarestatistics.humanservices.gov.au/statistics/pbs_group.jsp.

[24] Australian Bureau of Statistics , “National, State and Territory Population,” accessed January 25, 2024, https://www.abs.gov.au/statistics/people/population/national‐state‐and‐territory‐population/latest‐release.

[25] Australian Government Department of Health and Aged Care , “The Pharmaceutical Benefits Scheme,” accessed January 17, 2024, https://www.pbs.gov.au/pbs/home.

[26] World Health Organization Collaborating Centre for Drug Statistics Methodology , “ATC/DDD Index 2024,” accessed January 17, 2024, https://atcddd.fhi.no/atc_ddd_index/.

[27] Australian Institute of Health and Welfare , “Cancer Data in Australia,” accessed August 26, 2024, https://www.aihw.gov.au/reports/cancer/cancer‐data‐in‐australia/.

[28] Australian Bureau of Statistics , “Profile of Australia's Population,” accessed August 27, 2024, https://www.aihw.gov.au/reports/australias‐health/profile‐of‐australias‐population.

[29] World Health Organization , “Global Cancer Burden Growing, Amidst Mounting Need for Services,” accessed November 2, 2024, https://www.who.int/news/item/01‐02‐2024‐global‐cancer‐burden‐growing‐‐amidst‐mounting‐need‐for‐services. PMC11115397 38438207

[30] F. H. Alyami and J. J. Guo , “Trends in Utilization, Prices, and Spending for Oral Medications for Breast Cancer Treatment in the US Medicaid Population,” American Health & Drug Benefits 16, no. 1 (2023): 1–12, https://www.ahdbonline.com/issues/2023/june‐2023‐vol‐16‐no‐1/trends‐in‐utilization‐prices‐and‐spending‐for‐oral‐medications‐for‐breast‐cancer‐treatment‐in‐the‐us‐medicaid‐population‐2008‐2019.

[31] R. Roskoski, Jr. , “Properties of FDA‐Approved Small Molecule Protein Kinase Inhibitors: A 2024 Update,” Pharmacological Research 200 (2024): 107059, 10.1016/j.phrs.2024.107059. 38216005

[32] National Rural Health Alliance , “Cancer in Rural Australia,” accessed November 15, 2024, https://www.ruralhealth.org.au/sites/default/files/publications/nrha‐cancer‐factsheet‐july2022.pdf.

[33] Australian Bureau of Statisics , “Main Structure and Greater Capital City Statistical Areas: Australian Statistical Geography Standard (ASGS) Edition 3,” accessed November 4, 2024, https://www.abs.gov.au/statistics/standards/australian‐statistical‐geography‐standard‐asgs‐edition‐3/jul2021‐jun2026/main‐structure‐and‐greater‐capital‐city‐statistical‐areas.

[34] Australian Bureau of Statistics , “Remoteness Areas,” accessed November 4, 2024, https://www.abs.gov.au/statistics/standards/australian‐statistical‐geography‐standard‐asgs‐edition‐3/jul2021‐jun2026/remoteness‐structure/remoteness‐areas.

[35] L. Mellish , E. A. Karanges , M. J. Litchfield , et al., “The Australian Pharmaceutical Benefits Scheme Data Collection: A Practical Guide for Researchers,” BMC Research Notes 8 (2015): 634, 10.1186/s13104-015-1616-8. 26526064 PMC4630883

[36] J. P. Richmond , M. G. Kelly , A. Johnston , et al., “A Community‐Based Advanced Nurse Practitioner‐Led Integrated Oncology Care Model for Adults Receiving Oral Anticancer Medication: A Pilot Study,” Pilot and Feasibility Studies 10, no. 1 (2024): 46, 10.1186/s40814-024-01461-z. 38424625 PMC10902979

[37] M. H. A. Dennis , M. Johnson , J. Soggee , et al., “Cross‐Sectional Census Survey of Patients With Cancer Who Received a Pharmacist Consultation in a Pharmacist Led Anti‐Cancer Clinic,” Journal of Cancer Education 37, no. 5 (2022): 1553–1561, 10.1007/s13187-022-02196-2. 35867307 PMC9305046

[38] M. W. Mawhinney , J. Warden , and N. Stoner , “The Oral Education Clinic: A Pharmacist‐ and Nurse‐Led Clinic to Support Patients Starting Oral Systemic Anti‐Cancer Treatments,” Journal of Oncology Pharmacy Practice 25, no. 2 (2019): 449–453, 10.1177/1078155217727820. 28841100

